# Percutaneous Exposure Incidents of the Health Care Personnel in a Newly Founded Tertiary Hospital: A Prospective Study

**DOI:** 10.1371/journal.pone.0000194

**Published:** 2007-02-07

**Authors:** Matthew E. Falagas, Ioannis Karydis, Ilektra Kostogiannou

**Affiliations:** 1 Alfa Institute of Biomedical Sciences (AIBS), Athens, Greece; 2 Employee Health Clinic, Henry Dunant Hospital, Athens, Greece; 3 Department of Medicine, Tufts University School of Medicine, Boston, Massachusetts, United States of America; 4 Department of Nursing, Henry Dunant Hospital, Athens, Greece; Finnish Institute of Occupational Health, Finland

## Abstract

**Background:**

Percutaneous exposure incidents (PEIs) and blood splashes on the skin of health care workers are a major concern, since they expose susceptible employees to the risk of infectious diseases. We undertook this study in order to estimate the overall incidence of such injuries in a newly founded tertiary hospital, and to evaluate possible changes in their incidence over time.

**Methodology/Principal Findings:**

We prospectively studied the PEIs and blood splashes on the skin of employees in a newly founded (October 2000) tertiary hospital in Athens, Greece, while a vaccination program against hepatitis B virus, as well as educational activities for avoidance of injuries, were taking place. The study period ranged from October 1, 2002 to February 28, 2005. Serologic studies for hepatitis B (HBV) and C virus (HCV) as well as human immunodeficiency virus (HIV) were performed in all injured employees and the source patients, when known. High-titer immunoglobulin (250 IU anti-HBs intramuscularly) and HBV vaccination were given to non-vaccinated or previously vaccinated but serologically non-responders after exposure. Statistical analysis of the data was performed using Mc Nemar's and Fisher's tests. 60 needlestick, 11 sharp injuries, and two splashes leading to exposure of the skin or mucosa to blood were reported during the study period in 71 nurses and two members of the cleaning staff. The overall incidence (percutaneous injuries and splashes) per 100 full-time employment-years (100 FTEYs) for high-risk personnel (nursing, medical, and cleaning staff) was 3.48, whereas the incidence of percutaneous injuries (needlestick and sharp injuries) alone per 100 FTEYs was 3.38. A higher incidence of injuries was noted during the first than in the second half of the study period (4.67 versus 2.29 per 100 FTEYs, p = 0.005). No source patient was found positive for HCV or HIV. The use of high-titer immunoglobulin after adjustment for the incidence of injuries was higher in the first than in the second half of the study period, although the difference was not statistically significant [9/49 (18.37%) vs 1/24 (4.17%), p = 0.15].

**Conclusions/Significance:**

Our data show that nurses are the healthcare worker group that reports most of PEIs. Doctors did not report such injuries during the study period in our setting. However, the possibility of even relatively frequent PEIs in doctors cannot be excluded. This is due to underreporting of such events that has been previously described for physicians and surgeons. A decrease of the incidence of PEIs occurred during the operation of this newly founded hospital.

## Introduction

Percutaneous exposure incidents (PEIs) (needlestick, sharp injuries, as well as splashes leading to exposure of the skin or mucosa to blood) are a potential mode of exposure to - and transmission of blood-borne infectious diseases among healthcare workers. Such injuries are a major concern in hospitals even in developed countries such as the US [1], [2]. According to the Centers for Disease Control and Prevention (CDC) approximately 600,000 health care workers in the United States experience exposures to blood each year [3]. These may occur in the emergency departments, in the operating room, in the radiology or other departments and may be related to faulty needle insertion techniques, needle recapping, or incautious disposal of contaminated needles and sharps [2], [4], [5]. Needlestick and sharp injuries may be combined with failure to use appropriate barrier garments (e.g. hand gloves of proper size). PEIs may increase hepatitis B virus (HBV), hepatitis C virus (HCV), and human immunodeficiency virus (HIV) transmission risk in the healthcare setting as has been thoroughly reviewed in the literature and postexposure prophylaxis, when available, is therefore recommended [6].

We performed a prospective study to estimate the incidence of PEIs of high-risk groups of employees in a newly founded tertiary hospital. Our objectives were to calculate the incidence of such events and to identify possible changes in their incidence over time.

## Methods

We performed a prospective study of PEI's in the healthcare setting of a newly founded hospital. The Infection Control Committee of the hospital approved the collection and analysis of the data.

We systematically recorded all PEIs reported by the healthcare personnel of “Henry Dunant” hospital during a period of 2 years and 5 months (1^st^ October 2002 to 28^th^ February 2005). “Henry Dunant” hospital is a tertiary hospital in Athens, Greece that started to receive inpatients in October 2000. It has a total of 450 beds and employed during the study period (10/2002–02/2005) 1,411 persons, among whom there are 252 doctors, 615 nurses, and 544 administrative employees and others. Upon emergence of each new incident, the infection control nurse (or her replacement) was immediately notified. She documented each incident and referred the employee for further evaluation and treatment. Specifically, testing of the serologic status for HBV [by measuring HBV surface antigen (HbsAg) and antibody (anti-HBs)], HCV (by measuring HCV antibody) as well as HIV (by measuring HIV antibodies by ELISA testing and verified by Western Blot testing) of both the patient and the healthcare personnel involved in these accidents was performed, based on a protocol approved by the Infection Control Committee of the hospital. Testing of the serologic status of the source patient was not done in a small number of cases, when we could not clarify the source patient. The procedure enabled us to evaluate the risk of blood-borne disease transmission in each accident. Moreover, the infection control nurse recorded data about the management that was individualized according to evaluation of risk and immunization status for HBV including the treatment given, if any. The general treatment rule applied was that post-exposure prophylaxis with high-titer hyperimmune immunoglobulin (250 IU anti-HBs intramuscularly) and HBV vaccination should be given to unvaccinated or previously vaccinated but serologically non-responders (anti-HBs<10 mIU/ml) if the source patient was HBsAg seropositive, while no treatment should be given to previously vaccinated persons with adequate anti-HBs response (anti-HBs>or equal to 10 mIU/ml).

Educational and training programs for the healthcare workers were implemented throughout the study period aiming at reduction of injuries. These programs included a series of lectures on the dangers of blood-borne infectious diseases such as HBV, HCV and HIV and correct practices to minimize the risk of exposure to these infectious agents (voluntary participation). Moreover, an obligatory, introductory 2-hour tutorial focused on avoidance of PEI practices was offered to all nursing staff. In addition, written instructions regarding the handling of sharp needles and other instruments were distributed to the hospital nursing and medical personnel. Also, the infection control nurse discussed in detail the risks related to exposure to blood with the nurses of all nursing units of the hospital. Vaccination for HBV was offered to unvaccinated health care workers from the start of the operation of the hospital, and thus throughout the study period.

Statistical analysis of the data was performed using Mc Nemar's and Fisher's tests.

## Results

A total of 73 needlestick, sharp injuries, and splashes carrying the potential risk of blood-borne disease transmission in the healthcare personnel were reported during the study period. These involved 73 healthcare workers [nurses (n = 71) and cleaning staff (n = 2)]. Physicians did not report any PEI. The distribution of PEIs during the study period is presented in Table 1. Seven needlestick injuries were reported from 1^st^ October 2002 to 31 December 2002; 30 needlestick, 10 sharp injuries, and 2 cases of splashes were reported during 2003; 20 needlestick and 1 sharp injury was reported during 2004. Moreover, 3 needlestick injuries were reported from 1 January 2005 to 28 February 2005. The overall incidence (percutaneous injuries and splashes) per 100 full-time employment-years (100 FTEYs) for high-risk personnel (nursing, medical, and cleaning staff) was 3.48 whereas percutaneous injuries incidence per 100 FTEYs was 3.38 (the corresponding figures excluding doctors were 4.90 and 4.76 per 100 FTEYs, respectively). We also broke the study period into seven consecutive periods (from the beginning to the end of the study); the corresponding incidence rates for PEI's in nurses were 3.1, 2.4, 1.6, 1.0, 0.7, 1.8, and 1.0 per 100 FTEYs. The difference in the incidence of PEI between the first and second half of the time period studied was statistically significant (4.67 versus 2.29 per 100 FTEYs, p = 0.005 by Mc Nemar's test).

**Table 1:** Distribution of percutaneous exposure incidents during the study period (29 months).

	Females	Males	PEI's* Females	PEI's* Males
**Nursing staff**	498	117	67	4
**Medical staff**	58	194	0	0
**Administrative and other staff**	376	168	2**	0

** PEIs = Percutaneous exposure incidents (includes needlestick and sharp injuries as splashes leading to exposure of the skin or mucosa to blood)

* Housekeeping staff

No patient was found positive for HCV or HIV. Twenty-nine of 73 healthcare workers who suffered an accident had not been previously immunized against HBV. Ten of them subsequently received both high-titer immunoglobulin and active immunization against HBV; overall use of high-titer immunoglobulin per injury in the first half of the time period studied was higher than in the second half of the period studied, although the difference was not statistically significant [9/49 (18.37%) vs 1/24 (4.17%), p = 0.15 by Fisher's test]. The rest received only vaccination against HBV because they were considered to have low risk for transmission, according to patient serologic status. Seroconversion for HBV did not occur in any of the healthcare workers involved in the injuries.

## Discussion

Our prospective study of PEIs of healthcare workers of a newly founded tertiary care hospital showed several noteworthy results. Nurses reported most needlestick and sharp injuries. This is in accordance with reports from other countries [3], [4]. Incidence of PEIs is likely related with usage of sharp instruments and nurses may be more likely than doctors (except surgeons, perhaps) to be handling sharps. It is interesting that, in our setting, doctors did not report PEIs during the study period. However, the possibility of even relatively frequent PEIs in physicians and surgeons cannot be excluded. This is due to the serious underreporting of such events by doctors, a fact that has been previously described [7]–[9].

The underreporting of PEIs by doctors may be related to their unwillingness to reveal the incidence or lack of motivation due to the belief that they can handle the issue themselves. The possibility of underreporting by various health worker groups is a major limitation of our study since it may be a confounding factor for estimating the overall incidence of PEIs in the healthcare setting and in comparing the incidence of PEIs among high-risk groups of health care personnel. It should be noted, however, that other studies also showed considerable underreporting of PEIs in the health care personnel. For example, the underreporting of sharp injuries ranged from 22% to 62% in a study of the health care personnel of several Iowa community hospitals [9]. Of interest, a clear inverse association between the frequency of recent injury and reporting likelihood was documented in that study. The overall incidence of reported injuries was 3.48/100 FTEYs in our study, which is comparable to the results of another Greek study [10] and other studies [11]–[14].

Another noteworthy finding of the study is that the number of PEIs declined by more than 50% in the second half period of the study. Our study did not have enough statistical power for a more sophisticated analysis regarding the trends of the incidence of PEIs during the study period. Also, the lack of analysis based on the age of the employees is a limitation of this study. Reduction of needlestick injuries in a period of five years has been previously reported in an academic health center [15]. This is in contrast to results from another tertiary hospital where the incidence of needlestick injuries increased in a period of ten years [16]. It must be noted, however, that a considerable proportion of the nurses in our newly founded hospital were young, newly hired, and with limited professional experience. Accumulating experience and ongoing education and training, including increased risk awareness of the health care personnel, may have contributed to the decline of the incidence of PEIs during the study period. Yet, another limitation of our study is that we did not quantify the contribution of better education and training throughout the study period in the reduction of the incidence of PEIs.

The measures taken during our study period were in general in accordance with the Centers for Disease Control and Prevention (CDC) recommendations for baseline source serologic testing for HBV, HCV, and HIV. In addition, post-exposure prophylaxis for HBV was given also according to the relevant CDC guidelines. It is fortunate that no source positive for HCV or HIV was identified [17].

The implementation of an ongoing vaccination program against HBV led to considerable reduction of use of high-titer immunoglobulin against HBV that is an expensive treatment. Other studies have shown that a vaccination program in healthcare workers against HBV is cost-effective, decreases the anxiety of an employee after needlestick and sharp injuries, and prevents the transmission of HBV after exposure in the majority of cases [18]–[20]. Apart from doctors, it is, therefore, important to persuade nurses and the rest of the high-risk healthcare personnel including ambulance workers and housekeepers, who are seronegative for HBV to receive the currently available HBV vaccines. Post-exposure prophylaxis has to be given after quick but careful risk assessment, according to the availability of specific treatment and criteria of cost-effectiveness.

Based on the relevant CDC guidelines on the important clinical problem of PEIs, immediate monitoring and serologic assessment of the patient and the healthcare worker involved in the accident should be performed promptly in order to estimate the risk of blood-borne infection transmission. Continuous monitoring, careful evaluation, and prompt treatment of PEI can minimize the risk of blood-borne infection transmission in the healthcare setting. Moreover, accumulation of experience, continuous education and training may aid in decreasing the incidence of PEIs in the health care settings.

### Conclusions

It can be concluded from our study that nurses are the health care group that reports most PEIs. Although speculative, it is possible that the decreasing incidence of PEI's observed during the time period studied was partly attributed to cumulative experience gained by the nurses in this newly founded hospital. Future studies should focus on the estimation of the specific effect of various preventive measures of PEIs (including the implementation of new relevant educational programs for the hospital staff). This is probably the best research methodology that will allow the calculation of the effect of such preventive measures, because once all measures are used in daily practice it is very difficult to randomise health care workers to a control condition.
